# Physical activity, cardiorespiratory fitness, and cardiovascular outcomes in individuals with atrial fibrillation: the HUNT study

**DOI:** 10.1093/eurheartj/ehaa032

**Published:** 2020-02-11

**Authors:** Lars E Garnvik, Vegard Malmo, Imre Janszky, Hanne Ellekjær, Ulrik Wisløff, Jan P Loennechen, Bjarne M Nes

**Affiliations:** Department of Circulation and Medical Imaging, Faculty of Medicine and Health Sciences, Norwegian University of Science and Technology, Medisinsk Teknisk Forskningssenter, PO Box 8905, 7491 Trondheim, Norway; Department of Circulation and Medical Imaging, Faculty of Medicine and Health Sciences, Norwegian University of Science and Technology, Medisinsk Teknisk Forskningssenter, PO Box 8905, 7491 Trondheim, Norway; Clinic of Cardiology, St. Olav’s Hospital, Prinsesse Kristinas gate 3, Postboks 8905, 7491 Trondheim, Norway; Department of Public Health and Nursing, Faculty of Medicine and Health Sciences, Norwegian University of Science and Technology, Medisinsk Teknisk Forskningssenter, PO Box 8905, 7491 Trondheim, Norway; Department of Neurology, Medical School, University of Pécs, Rét u. 2, 7623 Pécs, Hungary; Institute of Behavioural Sciences, Semmelweis University, Nagyvárad tér 4, H-1089 Budapest, Hungary; Stroke Unit, Department of Internal Medicine, St Olav’s Hospital, Harald Hardrådes gate 14, 7030 Trondheim, Norway; Department of Neuromedicine and Movement Science, Norwegian University of Science and Technology, Medisinsk Teknisk Forskningssenter, PO Box 8905, 7491 Trondheim, Norway; Department of Circulation and Medical Imaging, Faculty of Medicine and Health Sciences, Norwegian University of Science and Technology, Medisinsk Teknisk Forskningssenter, PO Box 8905, 7491 Trondheim, Norway; School of Human Movement & Nutrition Sciences, University of Queensland, St Lucia QLD 4072, Australia; Department of Circulation and Medical Imaging, Faculty of Medicine and Health Sciences, Norwegian University of Science and Technology, Medisinsk Teknisk Forskningssenter, PO Box 8905, 7491 Trondheim, Norway; Clinic of Cardiology, St. Olav’s Hospital, Prinsesse Kristinas gate 3, Postboks 8905, 7491 Trondheim, Norway; Department of Circulation and Medical Imaging, Faculty of Medicine and Health Sciences, Norwegian University of Science and Technology, Medisinsk Teknisk Forskningssenter, PO Box 8905, 7491 Trondheim, Norway; Clinic of Cardiology, St. Olav’s Hospital, Prinsesse Kristinas gate 3, Postboks 8905, 7491 Trondheim, Norway

**Keywords:** Exercise, Arrhythmias, Population, Cardiovascular disease

## Abstract

**Aims:**

Atrial fibrillation (AF) confers higher risk of mortality and morbidity, but the long-term impact of physical activity (PA) and cardiorespiratory fitness (CRF) on outcomes in AF patients is unknown. We, therefore, examined the prospective associations of PA and estimated CRF (eCRF) with all-cause mortality, cardiovascular disease (CVD) mortality, morbidity and stroke in individuals with AF.

**Methods and results:**

We followed 1117 AF patients from the HUNT3 study in 2006–08 until first occurrence of the outcomes or end of follow-up in November 2015. We used Cox proportional hazard regression to examine the prospective associations of self-reported PA and eCRF with the outcomes. Atrial fibrillation patients meeting PA guidelines had lower risk of all-cause [hazard ratio (HR) 0.55, 95% confidence interval (CI) 0.41–0.75] and CVD mortality (HR 0.54, 95% CI 0.34–0.86) compared with inactive patients. The respective HRs for CVD morbidity and stroke were 0.78 (95% CI 0.58–1.04) and 0.70 (95% CI 0.42–1.15). Each 1-metabolic equivalent task (MET) higher eCRF was associated with a lower risk of all-cause (HR 0.88, 95% CI 0.81–0.95), CVD mortality (HR 0.85, 95% CI 0.76–0.95), and morbidity (HR 0.88, 95% CI 0.82–0.95).

**Conclusion:**

Higher PA and CRF are associated with lower long-term risk of CVD and all-cause mortality in individuals with AF. The findings support a role for regular PA and improved CRF in AF patients, in order to combat the elevated risk for mortality and morbidity.

**See page 1476 for the editorial comment on this article (doi: 10.1093/eurheartj/ehaa204)**

## Introduction 

Atrial fibrillation (AF) is the most common sustained arrhythmia and is associated with increased rates of mortality and morbidity.^1^ The presence of AF is also associated with worse prognosis in patients with coronary heart disease and heart failure.^2^ ^,^ ^3^ There has been a progressive increase in the global prevalence of AF over the last decades, and it is estimated that the number of patients with AF will continue to rise considerably in the coming years.^4^ ^,^ ^5^ Moreover, AF is a complex disease to manage, with few effective treatment options available, placing substantial demands on the healthcare systems. While oral anticoagulation is a cornerstone therapy shown to reduce mortality in AF patients, interventions for rhythm control such as ablation and antiarrhythmic medication may reduce symptoms, but have less clear long-term mortality benefits.^6^ Hence, there is a need for cost-effective preventive measures and long-term management strategies to combat the future burden of AF.

There is a large body of evidence supporting the role of physical activity (PA) and cardiorespiratory fitness (CRF) in rehabilitation and treatment of cardiovascular disease (CVD) and prevention of premature mortality.^7^ ^,^ ^8^ Prospective studies have shown that a moderate PA level^9^ ^,^ ^10^ and higher measured^11^ or estimated CRF (eCRF)^12^ are linked to reduced incidence of AF, although high volumes of endurance training increase AF risk. In established AF, exercise and PA have been shown to benefit underlying conditions and potentially reduce AF burden, but the evidence for long-term impact on clinical events and mortality is sparse. Due to lack of evidence, there are no current guidelines on PA for patients with AF. A recent study, however, indicated lower rates of major adverse events after 1-year follow-up among AF patients reporting regular or intense PA compared with no activity.^13^

The aim of this study was, therefore, to explore the long-term impact of PA and eCRF on all-cause and CVD mortality and morbidity in individuals with AF.

## Methods

### Participants

This study included data from the 3rd wave of the Nord-Trøndelag Health Study (HUNT3), carried out in 2006–08. HUNT is a large, population-based cohort study conducted in the northern region of Trøndelag, Norway. All residents above 18 years of age were invited.

Participants with confirmed AF at baseline in HUNT3 were identified: (i) Through linkage to hospital discharge registers at the two hospitals in the region. Code I48 (AF/flutter) from the ICD 10th Revision was used to identify possible AF. (ii) All participants with self-reported cardiovascular or renal disease in HUNT3 questionnaires were asked if a doctor had told them that they had AF. (iii) For a subgroup included in a previous validation study (*n* = 16 247), diagnoses from primary care (ICPC code K78 AF/flutter) were also included. For persons with possible AF from at least one of the groups above, hospital medical records, including electrocardiograms (ECGs), for inpatient and outpatient visits were obtained and reviewed. For the subpopulation from the validation study, medical records from primary care were also reviewed. Diagnoses were validated by a cardiologist and two specialists in internal medicine using ECGs according to standard criteria. Individuals were not regarded as having AF if they only had an episode related to cardiac surgery, acute myocardial infarction or major haemodynamic instability. The validation process of AF diagnoses in this cohort is previously described in detail.^14^ As a sub-analysis, we included all participants without known AF in HUNT3. Further details about the total HUNT3 cohort profile are published elsewhere.^15^

The regional committee for medical and health research ethics approved the study. All participants gave an informed written consent before participating.

### Clinical and questionnaire-based variables

Clinical examinations included measurements of height, weight, waist circumference, blood pressure, resting heart rate, and blood samples. We defined body mass index (BMI) as weight divided by the square of the height in metres (kg/m^2^). Blood pressure and resting heart rate were measured three times at 1-min intervals using a Dinamap 845XT (Citikon, Tampa, USA), and the average of the 2nd and 3rd measurements was used. Self-reported data included information on PA, occupational status, smoking, alcohol, antihypertensive medication, and disease status, including CVD and diabetes.

### Ascertainment of exposures

Participants reported their PA levels by answering three questions about the frequency, intensity, and duration of exercise. Frequency was stated as ‘How often do you exercise?’, with the response options ‘Never’, ‘Less than once a week’, ‘Once a week’, ‘2–3 times a week’, or ‘Almost every day’. Intensity was stated as ‘How hard do you push yourself?’ with response options ‘I take it easy, I don’t get out of breath or break a sweat’, ‘I push myself until I’m out of breath and break into a sweat’, or ‘I practically exhaust myself’. Duration was stated as ‘How long does each session last?’ with response options ‘Less than 15 min’, ‘15–29 min’, ‘30 min to 1 h’, or ‘More than 1 h’. The PA questionnaire has previously been validated.^16^

We calculated the average minutes of weekly PA by multiplying frequency and median duration per session. Minutes were combined with intensity, with the two highest-intensity categories combined, to classify participants into three groups according to the general PA recommendations^17^. (1) *Inactive*, reflecting no PA or less than once a week; (2) *below*, reflecting <150 min of moderate intensity or 75 min of vigorous intensity per week; (3) *at or above*, ≥150 min of moderate intensity or ≥75 min of vigorous intensity. We also performed stratified analyses by moderate vs. vigorous intensity across three categories of total PA time (<75, 75–149, and ≥150 min per week).

To estimate CRF (peak oxygen uptake, VO_2peak_), we used a non-exercise prediction model previously published by our group^18^ and validated in a sample of 635 individuals with AF and objective VO_2peak_ (Supplementary material online). The model was sex-specific and based on age, waist, resting heart rate, and PA. Once eCRF was calculated individually, we divided the participants in sex-specific quartiles within 10-year age groups (<40, 40–49, 50–59, 60–69, and ≥70 years) and combined them to form quartiles for the whole cohort, as previously recommended.^19^

### Follow-up and ascertainment of outcomes

We linked HUNT3 data to the Norwegian Cause of Death Registry and Norwegian Patient Registry to study the association between PA and eCRF on the following four outcomes: all-cause mortality, CVD mortality; defined as all deaths with CVD as the underlying cause (ICD-10, I00–I99), CVD morbidity; as a composite endpoint including first onset of myocardial infarction (ICD-10, I21), heart failure (ICD-10, I50), or haemorrhagic or ischaemic stroke (ICD-10, I61, I63) and stroke. The follow-up period lasted from baseline to first occurrence of the outcomes or end of follow-up in November 2015, whichever came first. We censored participants at the time of death from other causes than the outcome of interest.

### Statistics

Descriptive data are presented as means ± standard deviations (SD) for continuous variables and numbers and percentages (%) for categorical variables. We used Cox proportional hazards regression with 95% confidence intervals (CIs) to study the prospective association between PA, and eCRF, and each outcome, respectively. All models were developed with attained age as time scale, and the proportional hazards assumption was tested with Schoenfeld residuals and no violation of the assumption was found. We then constructed two models. Model 1 was adjusted for attained age and sex. Model 2 was further adjusted for CVD, smoking habits, alcohol intake, occupational status, and BMI. For eCRF, BMI was not included, because waist is included in the eCRF algorithm and further including BMI would potentially leads to severe collinearity. Hazard ratios (HRs) with 95% CIs for the outcomes are presented according to PA categories, per metabolic equivalent task (MET) and by quartiles of eCRF. We constructed Kaplan–Meier curves to present event-free survival probability according to PA and eCRF categories. As a sub-analysis, we examined the combined associations of AF vs. non-AF and PA using the full HUNT3 cohort. Inactive non-AF participants were the reference, and analyses were adjusted for diabetes and hypertension in addition to the covariates in Model 2.

Several sensitivity analyses were conducted to test the robustness of our results. First, we examined potential effect modification by investigating the association of PA and eCRF with the outcomes within subgroups of sex, age, BMI, and self-reported CVD at baseline (myocardial infarction, heart failure, angina pectoris, and stroke). Second, we excluded the first 2 years of follow-up in the main analyses to reduce the possibility that our results were affected by reversed causality. Third, because resting heart rate is included in the eCRF algorithm and could potentially be affected by AF episodes during measurements, we performed a sensitivity analysis adjusting for resting heart rate. Fourth, we performed analyses further adjusting for a modified CHA_2_DS_2_VASc score. Lastly, we further adjusted for, and examined effect modification by, AF subtype (paroxysmal, persistent, permanent) and use of beta-blockers in a subset of 477 participants from which this information was available. Analyses were performed using STATA 15 (StataCorp, TX, USA).

## Results

### Participants and descriptive data

The total adult population of 93 860 men and women in Nord-Trøndelag county were invited to HUNT3, of whom 50 802 responded (54.1%). After excluding 321 participants with missing data, we included 1117 participants with confirmed AF. Baseline characteristics of the AF population according to PA are presented in *Table 1*. There were 347 (31%) women and 770 (69%) men. Women were 73.1 (±10.8) and men were 70.1 (±10.2) years old. Characteristics of the general population of 42 375 participants without known AF at baseline or during follow-up are presented in Supplementary material online, *Table S4*.

**Table 1:** Baseline characteristics of atrial fibrillation patients according to general physical activity recommendations

	Inactive	Not meeting	Meeting
No. of participants	306 (27.4)	447 (40.0)	364 (32.6)
Sex			
Women	118 (38.6)	149 (33.3)	80 (22.0)
Men	188 (61.4)	298 (66.7)	284 (78.0)
Age (years)	72.9 ± 10.7	71.4 ± 10.0	69.0 ± 10.5
Height (cm)	170.3 ± 10.3	171.9 ± 9.	174.2 ± 8.7
Weight (kg)	85.8 ± 17.0	83.5 ± 15.1	83.1 ± 14.8
Waist (cm)	103.0 ± 12.6	99.2 ± 11.7	97.1 ± 11.5
Body mass index (kg/m^2^)	29.5 ± 4.9	28.2 ± 4.3	27.3 ± 4.1
Systolic blood pressure (mmHg)	134.1 ± 21.1	135.4 ± 20.3	134.0 ± 20.3
Diastolic blood pressure (mmHg)	74.7 ± 13.0	75.6 ± 12.6	76.2 ± 11.6
Resting heart rate (b.p.m.)	66.1 ± 12.0	65.9 ± 12.8	64.8 ± 12.6
eCRF (mL/kg/min)	27.4 ± 6.6	29.7 ± 6.2	34.8 ± 6.8
eCRF (METs)	7.8 ± 1.9	8.5 ± 1.8	10.0 ± 1.9
CHA_2_DS_2_VASc risk score			
Low-moderate	60 (19.7)	118 (26.5)	118 (32.4)
High	245 (80.3)	328 (73.5)	246 (67.6)
Smoking status			
Non-smoker	252 (82.4)	373 (83.5)	317 (87.1)
Daily smoker	36 (11.8)	45 (10.1)	25 (6.9)
Occasional	18 (5.9)	29 (6.5)	22 (6.0)
Alcohol use^a^	179 (58.5)	301 (67.3)	282 (77.5)
Hypertension^b^	245 (80.1)	345 (77.2)	237 (65.1)
Heart failure	65 (21.2)	66 (14.8)	41 (11.3)
Myocardial infarction	51 (16.7)	70 (15.7)	47 (12.9)
Stroke	42 (13.7)	58 (13.0)	30 (8.2)
Diabetes	56 (18.4)	43 (9.6)	23 (6.3)
AF subtype (%)^c^			
Paroxysmal	37.4	42.2	48.1
Persistent	18.3	14.3	13.5
Permanent	44.3	43.5	38.5
Beta-blocker use (%)^c^	68.7	64.6	46.2

Data are presented as means ± SD or No. (percentages).

eCRF, estimated cardiorespiratory fitness; MET, metabolic equivalent task; PA, physical activity.

a Alcohol use last 2 weeks.

b Systolic blood pressure ≥140 mmHg and/or diastolic blood pressure ≥90 mmHg and/or use of antihypertensive medication.

c *n* = 477.

### All-cause and cardiovascular disease mortality

Survival probabilities for all-cause and CVD mortality according to categories of PA and eCRF are presented in *Take home figure* and Supplementary material online, *Figure S1*. Hazard ratios with 95% CIs for all-cause and CVD mortality according to PA and eCRF are shown in *Table 2*. Atrial fibrillation individuals meeting the general PA recommendations had 45% lower risk for all-cause mortality compared with inactive (HR 0.55, 95% CI 0.41–0.75). The risk reduction of all-cause mortality per MET higher eCRF was 12% (HR 0.88, 95% CI 0.81–0.95) and participants with highest eCRF levels had 36% lower risk than those with lowest (HR 0.64, 95% CI 0.47–0.89).

**Table 2:** Hazard ratios with 95% confidence intervals for all-cause and CVD mortality according to physical activity recommendations and estimated cardiorespiratory fitness

	*n*	Events	Model 1^a^	Model 2^b^
All-cause mortality				
PA recommendations				
Inactive	306	130	1 (ref.)	1 (ref.)
Not meeting	447	139	0.78 (0.61–0.99)	0.77 (0.60–0.99)
Meeting	364	75	0.57 (0.42–0.76)	0.55 (0.41–0.75)
			*P*-trend <0.001	*P*-trend <0.001
eCRF^c^				
Per MET	1117	344	0.88 (0.82–0.95)	0.88 (0.81–0.95)
Quartile 1	284	109	1.0 (ref.)	1.0 (ref.)
Quartile 2	276	100	0.72–1.24	0.92 (0.70–1.21)
Quartile 3	282	75	0.77 (0.57–1.03)	0.75 (0.56–1.01)
Quartile 4	275	60	0.67 (0.49–0.92)	0.64 (0.47–0.89)
			*P*-trend 0.006	*P*-trend 0.003
CVD mortality				
PA recommendations				
Inactive	306	64	1 (ref.)	1 (ref.)
Not meeting	447	68	0.78 (0.56–1.11)	0.87 (0.61–1.24)
Meeting	364	30	0.49 (0.31–0.76)	0.54 (0.34–0.86)
			*P*-trend 0.002	*P*-trend 0.012
eCRF^c^				
Per MET	1117	162	0.85 (0.76–0.95)	0.85 (0.76–0.95)
Quartile 1	284	55	1 (ref.)	1 (ref.)
Quartile 2	276	49	0.92 (0.62–1.36)	0.91 (0.61–1.36)
Quartile 3	282	31	0.63 (0.40–0.98)	0.62 (0.40–0.98)
Quartile 4	275	27	0.62 (0.39–0.98)	0.61 (0.38–0.98)
			*P*-trend 0.012	*P*-trend 0.012

Data are presented as hazard ratios (95% confidence intervals).

CI, confidence interval; CVD, cardiovascular disease; MET, metabolic equivalent task; PA, physical activity.

a Model 1 adjusted for sex and age by including attained age as the time scale.

b Model 2 adjusted for model 1 + body mass index, CVD, smoking, alcohol, and occupational status.

c Model 2 adjusted for model 2—body mass index.

For CVD mortality, participants meeting PA recommendations had 46% lower risk than inactive (HR 0.54, 95% CI 0.34–0.86). Each MET higher eCRF was associated with 15% reduced CVD mortality risk (HR 0.85, 95% CI 0.76–0.95), and those in the highest eCRF quartile had 39% lower risk compared with those in the lowest category (HR 0.61, 95% CI 0.38–0.98).

### Cardiovascular disease morbidity and stroke

Physical activity level according to recommendations was associated with 22% lower risk of CVD morbidity (HR 0.78, 95% CI 0.58–1.04, *Table 3*) and 30% lower risk of stroke (HR 0.70, 95% CI 0.42–1.15). Each 1-MET higher eCRF was associated with 12% lower risk of CVD morbidity (HR 0.88, 95% CI 0.82–0.95) and 7% lower risk of stroke (HR 0.93, 95% CI 0.83–1.05). Moreover, those in the highest eCRF quartile had 31% lower risk of morbidity and 35% lower risk of stroke, respectively, compared with those in the lowest quartile (HR 0.69, 95% CI 0.51–0.93 and 0.65, 95% CI 0.39–1.11).

**Table 3:** Hazard ratios with 95% confidence intervals for cardiovascular disease morbidity and stroke according to physical activity recommendations and estimated cardiorespiratory fitness

	*n*	Events	Model 1^a^	Model 2^b^
CVD morbidity				
PA recommendations				
Inactive	306	108	1 (ref.)	1 (ref.)
Not meeting	447	155	1.00 (0.78–1.28)	0.98 (0.76–1.27)
Meeting	364	95	0.78 (0.59–1.04)	0.78 (0.58–1.04)
			*P*-trend 0.088	*P*-trend 0.096
eCRF^c^				
Per MET	1117	358	0.89 (0.83–0.96)	0.88 (0.82–0.95)
Quartile 1	284	107	1 (ref.)	1 (ref.)
Quartile 2	276	88	0.84 (0.63–1.11)	0.81 (0.61–1.08)
Quartile 3	282	88	0.86 (0.65–1.15)	0.86 (0.65–1.15)
Quartile 4	275	75	0.72 (0.53–0.96)	0.69 (0.51–0.93)
			*P*-trend 0.042	*P*-trend 0.028
Stroke				
PA recommendations				
Inactive	306	42	1 (ref.)	1 (ref.)
Not meeting	447	58	0.97 (0.65–1.45)	0.99 (0.66–1.49)
Meeting	364	30	0.68 (0.42–1.10)	0.70 (0.42–1.15)
			*P*-trend 0.126	*P*-trend 0.177
eCRF^c^				
Per MET	1117	130	0.93 (0.83–1.05)	0.93 (0.83–1.05)
Quartile 1	284	37	1 (ref.)	1 (ref.)
Quartile 2	276	41	1.18 (0.76–1.85)	1.22 (0.77–1.91)
Quartile 3	282	29	0.86 (0.53–1.40)	0.87 (0.53–1.42)
Quartile 4	275	23	0.66 (0.39–1.11)	0.65 (0.39–1.11)
			*P*-trend 0.071	*P*-trend 0.069

CVD, cardiovascular disease; eCRF, estimated cardiorespiratory fitness; MET, metabolic equivalent task; PA, physical activity.

a Model 1 adjusted for sex and age by including attained age as the time scale.

b Model 2 adjusted for model 1 + body mass index, CVD, smoking, alcohol and occupational status.

c Model 2 adjusted for model 2—body mass index.

### Moderate *vs.* vigorous intensity physical activity

**Figure 1:**
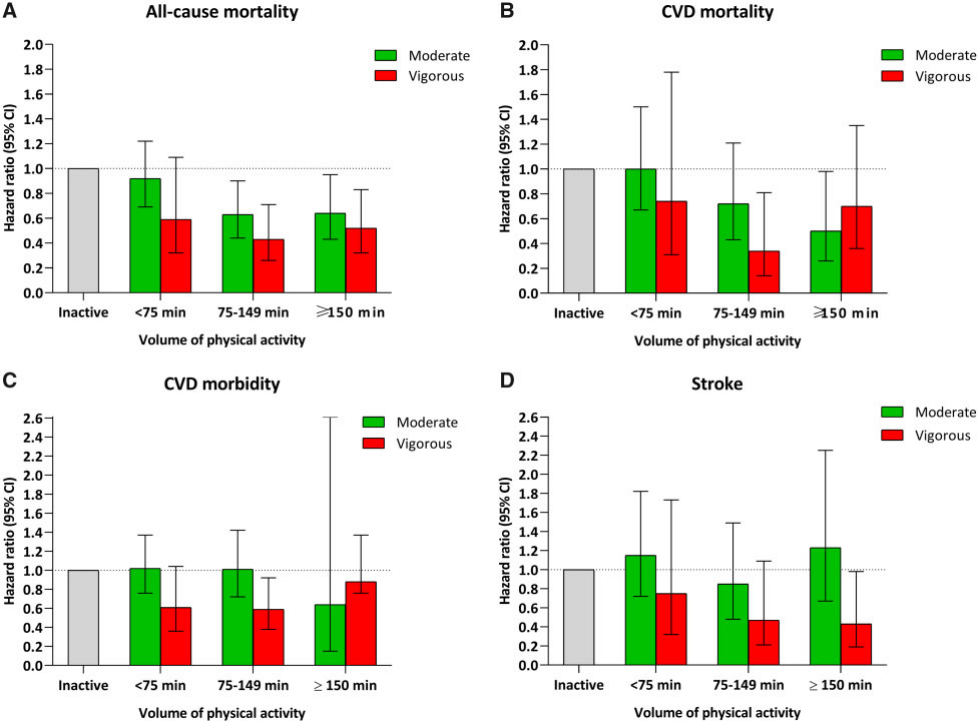
(*A–D*) Hazard ratio and 95% confidence interval for all-cause (*A*), cardiovascular disease mortality (*B*), cardiovascular disease morbidity (*C*), and stroke (*D*) across self-reported volume of physical activity, stratified by intensity. Adjusted for attained age, sex, body mass index, cardiovascular disease, smoking, alcohol, and occupational status.

Overall, the risk of each outcome was slightly lower among those reporting vigorous intensity compared with moderate across weekly time spent on PA (*Figure 1*). For CVD mortality, however, those reporting ≥150 min/week of vigorous intensity had 30% lower risk compared with inactive (HR 0.70, 95% CI 0.36–1.35), while those reporting moderate intensity had 50% lower risk (HR 0.50, 95% CI 0.26–0.98).

### Atrial fibrillation individuals *vs.* the general population

**Figure 2:**
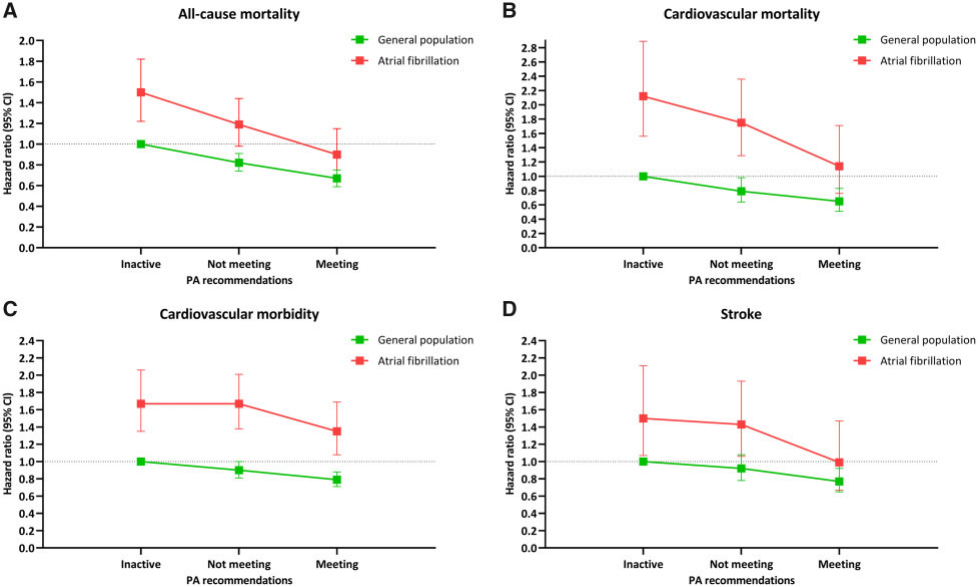
(*A–D*) Hazard ratio and 95% confidence interval for all-cause (*A*), cardiovascular disease mortality (*B*), cardiovascular disease morbidity (*C*), and stroke (*D*) across physical activity level in individuals with and without atrial fibrillation. Adjusted for attained age, sex, body mass index, cardiovascular disease, diabetes, hypertension, smoking, alcohol, and occupational status.

Compared with inactive participants without AF from the general population, those with AF who were inactive or not meeting recommendations, respectively, had consistently higher risk of each outcome (*Figure 2* and Supplementary material online, *Table S5*). Atrial fibrillation individuals who met PA recommendations, however, did not have a considerably higher risk of neither all-cause mortality (HR 0.90, 95% CI 0.70–1.15), CVD mortality (HR 1.14, 95% CI 0.76–1.71), nor stroke (HR 0.99, 95% CI 0.67–1.47), compared with the inactive non-AF group.

**Take home figure:**
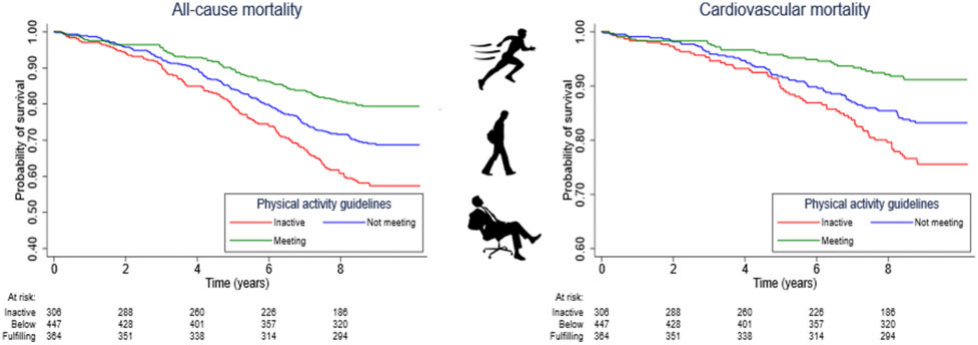
Survival probabilities for all-cause and CVD mortality according to physical activity level among individuals with atrial fibrillation.

### Sub- and sensitivity analyses

Physical activity was associated with lower risk of CVD mortality and morbidity in participants with BMI <30, but not among obese (Supplementary material online, *Figure S2b, c*). Moreover, each 1-MET higher eCRF was associated with lower risk of CVD morbidity in men, but not women (Supplementary material online, *Figure S3c*). Otherwise, the results were consistent within subgroups of sex, age, BMI, and CVD.

Excluding the first 2 years of the follow-up did not affect the point estimates to any great extent, for any outcome (Supplementary material online, *Table S1*). Furthermore, additional adjustment for resting heart rate did not change the associations between eCRF and outcomes (HRs 0.90, 95% CI 0.83–0.99; 0.88, 95% CI 0.77–0.99; 0.89, 95% CI 0.80–0.98; and 0.88, 95% CI 0.77–1.01 for all-cause, CVD mortality, morbidity, and stroke, respectively, per 1-MET higher eCRF). Neither adjusting for CHA_2_DS_2_VASc risk score did change the associations between PA or eCRF, respectively, and the outcomes (Supplementary material online, *Table S2*). Lastly, further adjustment for use of beta-blockers and AF type, respectively, in a subgroup did not considerably affect the associations between PA, eCRF, and all-cause mortality (Supplementary material online, *Table S5*). There was no evidence of effect modification by permanent vs. non-permanent AF or beta-blockers, although the power to detect subgroup associations was low (Supplementary material online, *Figure S4*).

## Discussion

In this study, we demonstrate that PA and eCRF were inversely associated with long-term all-cause and cardiovascular mortality risk in individuals with confirmed AF. Furthermore, we show that higher eCRF is related to lower risk of CVD morbidity. Similar trends were observed for stroke, although the precision of the estimates was lower, hence precluding any firm conclusions.

The beneficial impact of PA and CRF on CVD incidence and mortality is well documented in healthy populations.^20–22^ Also, the association of PA and CRF with AF incidence has been extensively studied the last decades.^9^ ^,^ ^23–25^ However, studies on the long-term impact of PA and CRF on adverse cardiovascular outcomes in AF patients have been lacking. Our findings are of importance given the lack of specific exercise recommendations for AF patients, despite that this group often possess a high burden of CVD risk factors and comorbidities that would generally benefit from PA interventions. Common AF symptoms, such as palpitations, exercise intolerance, and dyspnoea, may also have prevented many patients from engaging in PA.

Atrial fibrillation has been consistently related to increased rates of mortality and morbidity,^26^ as well as worse prognosis in patients with CVD.^2^ ^,^ ^3^ Notably, AF participants in our study who were active according to PA recommendations, had highly attenuated risk of all outcomes, and relative risks of all-cause, CVD mortality, and stroke comparable with the general population without AF. In a very distinct population of endurance-trained skiers, Svedberg *et al*.^27^ reported halved mortality incidence and lower stroke risk in skiers compared with non-skiers with AF. Still, skiers with AF had considerably higher stroke incidence than non-skiers without AF.

Our results are in line with a study by Proietti *et al*.^13^ who followed 2415 AF patients for 1 year and demonstrated lower rates of all-cause mortality among physically active compared with inactive AF patients. Furthermore, PA was inversely associated with a composite outcome of CVD mortality and any thromboembolic event/bleeding.

Although direct causality cannot be implied by this study, there are several potential mechanisms by which PA and CRF could reduce risk of adverse outcomes in AF. First, it is well established that PA improves the CVD risk factor profile,^8^ which may contribute to reduced long-term risk of ischaemic heart diseases and mortality in AF patients. Also, the benefits of high CRF levels are reasonable, since CRF reflects physical function and capacity and is consistently linked to lower CVD and mortality, independent of traditional risk factors, and in both healthy and CVD patients.^7^ Furthermore, PA and CRF may induce favourable effects on several of the pathophysiological mechanisms that contribute to the development and maintenance of AF. Pathak *et al*.^28^ examined the role of CRF, and CRF gain, on rhythm control in obese AF patients after a structured weight management programme including exercise. They demonstrated that after a mean follow-up of 49 months, high baseline CRF was related to less AF recurrence, and that each MET increase was associated with 13% reduced risk of AF recurrence, irrespective of weight loss. Moreover, our group has previously shown that 12 weeks of aerobic interval training in non-permanent AF patients increased VO_2peak_ by ∼1 MET accompanied by a reduced AF burden compared with controls.^29^ Although it is unclear whether AF independently contributes to poorer patient outcomes or whether AF is just a marker for other underlying conditions,^30^ a reduced AF burden could potentially also limit structural and electrophysiological remodelling of the heart in the long term, leading to less cardiac strain and subsequent lower risk of events. Further studies are, however, encouraged to determine the mechanistic pathways through which PA and CRF may act to reduce long-term risk in AF.

### Strengths and limitations

The main strengths of this study are the prospective design, the long-term follow-up period, and the linkage to national mandatory outcome registries. Furthermore, we used validated^14^ AF diagnoses from hospital registers and primary care to define our study population. However, there are several limitations to this study that needs to be addressed. First, our data do not establish a causal relationship between PA/eCRF and the outcomes, although the long follow-up, and similar results after excluding the first 2 years, reduce the possibility of reverse causality. Despite adjustment for a wide range of covariates, including several chronic diseases, we cannot exclude the possibility of residual confounding. We neither had data to explore potential effect modification by underlying aetiology, such as AF induced by long-term endurance training. Second, PA was self-reported and CRF was estimated which may have led to exposure misclassification, but due to the prospective design of the study such misclassification is expected to be non-differential and thus leading to under- but not overestimation of effects. Third, we did not have data on AF burden and progression over time, which prevent us from delineate whether the risk reductions were modulated by reduced AF *per se*. Fourth, we did not have information on medication, such as use of anticoagulants at baseline or during follow-up. However, adjusting for CHA_2_DS_2_VASc score, as a possible surrogate for anticoagulant use, did not influence the effect estimates. Fifth, the fact that resting heart rate is incorporated in the eCRF calculation could be problematic in AF patients. However, a sensitivity analysis revealed no change in the estimates when adjusting for resting heart rate. Lastly, it is possible that not all AF cases were detected, and the relatively low proportion of women with AF is a limitation.

## Conclusions

In this prospective cohort study of 1117 individuals with AF, PA, and higher levels of eCRF were associated with lower risk of all-cause and CVD mortality and morbidity. Our findings, therefore, support a role for regular PA and improved CRF in AF patients, in order to combat the elevated risk for mortality and morbidity.

## Supplementary Material

ehaa032_Supplementalry_Material
