# Supplementary material for: Physical activity, cardiorespiratory fitness, and cardiovascular outcomes in individuals with atrial fibrillation: the HUNT study
Source: Eur Heart J. 2020 Feb 11;41(15):1467–75. doi: 10.1093/eurheartj/ehaa032 (PMC7320825; doi:10.1093/eurheartj/ehaa032)
Supplement: ehaa032_Supplementalry_Material [file eurheartj_41_15_1467_s5.docx]

**Supplemental material**

Supplemental methods

The algorithms used to calculate eCRF was validated in an external sample of participants with verified and/or self-reported AF from the HUNT4 Fitness Study (2017-18). A total of 652 AF participants had a valid measurement of peak oxygen uptake (VO_2peak_), by use of ergospirometry (Metalyzer, Cortex Biophysik). An individualized treadmill protocol to exhaustion was applied. Physical activity (PA) was self-reported using the same questionnaire as in HUNT3. Waist circumference was calculated by InBody 770 analyzer and RHR by the same protocol and equipment as in HUNT3. eCRF was calculated using the following algorithms for men and women, respectively:

Men: 100.27 - (0.296 x age) - (0.369 x waist) - (0.155 x resting heart rate) + (0.226 x PA-I).

Women: 74.74 - (0.247 x age) - (0.259 x waist) - (0.114 x resting heart rate) + (0.198 x PA-I).

Mean difference between eCRF and VO_2peak_ was calculated for the total sample, by sex, and by sex- and age-specific quartiles of eCRF. In the total sample, calculated eCRF was 1.7 mL/kg/min higher than measured VO_2peak._ The difference was 0.2 and 2.5 in women and men, respectively. Within quartiles of eCRF no difference was observed in the low-fit group (Q1, mean diff. -0.4), while eCRF slightly overestimated VO_2peak_ in Q_2-4_ (mean diff. 2.1, 2.8 and 2.4, respectively). Pearson correlation coefficients were used to assess overall association (r=0.79). To indicate the ability of eCRF to correctly classify individuals with AF into the correct quartile of VO_2peak_, we cross-tabulated age- and sex-specific quartiles of eCRF and VO_2peak_, respectively. In total 56.9% of participants in Q_1_ of eCRF were also classified into Q_1_ of VO_2peak_ and 61.4 of Q_4_ of eCRF were also classified into Q_4_ of VO_2peak_. Moreover, 88% and 84% of the participants in Q_1_ and Q_4_ of eCRF, respectively, were classified within the closest category.

| **Supplementary table 1.** Hazard ratios with 95% CIs for all-cause and CVD mortality and CVD morbidity according to PA guidelines and eCRF in AF patients with the first 2 years of follow-up excluded. | | | | | | | |
| --- | --- | --- | --- | --- | --- | --- | --- |
| **PA guidelines*** | **All-cause mortality** |  | **CVD mortality** |  | **CVD morbidity** |  | **Stroke** |
| Inactive | 1 (ref.) |  | 1 (ref.) |  | 1 (ref.) |  | 1 (ref.) |
| Not meeting | 0.76 (0.58-0.99) |  | 0.88 (0.60-1.29) |  | 1.04 (0.77-1.40) |  | 1.03 (0.65-1.63) |
| Meeting | 0.49 (0.35-0.69) |  | 0.47 (0.28-0.78) |  | 0.84 (0.59-1.19) |  | 0.76 (0.43-1.33) |
|  | P-trend <0.001 |  | P-trend 0.006 |  | P-trend 0.314 |  | P-trend 0.350 |
| **eCRF**† |  |  |  |  |  |  |  |
| Per MET | 0.87 (0.80-0.95) |  | 0.83 (0.74-0.94) |  | 0.88 (0.80-0.96) |  | 0.94 (0.82-1.07) |
| Quartile 1 | 1 (ref.) |  | 1 (ref.) |  | 1 (ref.) |  | 1 (ref.) |
| Quartile 2 | 0.89 (0.66-1.21) |  | 0.82 (0.53-1.25) |  | 0.80 (0.57-1.11) |  | 1.34 (0.81-2.20) |
| Quartile 3 | 0.76 (0.55-1.05) |  | 0.54 (0.33-0.87) |  | 0.72 (0.51-1.01) |  | 0.70 (0.39-1.25) |
| Quartile 4 | 0.59 (0.41-0.84) |  | 0.52 (0.31-0.87) |  | 0.68 (0.48-0.97) |  | 0.77 (0.44-1.36) |
|  | P-trend 0.002 |  | P-trend 0.003 |  | P-trend 0.024 |  | P-trend 0.147 |
| Data are presented as hazard ratios (95% confidence intervals).  CVD = cardiovascular disease; eCRF = estimated cardiorespiratory fitness; PA = physical activity ^*^PA adjusted for attained age, sex, BMI, CVD, smoking, alcohol and occupational status. †eCRF adjusted for attained age, sex, CVD, smoking, alcohol and occupational status. | | | | | | | |

| **Supplementary table 2.** Hazard ratios with 95% CIs for all-cause mortality, CVD mortality, CVD morbidity and stroke according to physical activity and estimated cardiorespiratory fitness. | | | | |
| --- | --- | --- | --- | --- |
| **PA guidelines*** | **All-cause mortality** | **CVD mortality** | **CVD morbidity** | **Stroke** |
| Inactive | 1 (ref.) | 1 (ref.) | 1 (ref.) | 1 (ref.) |
| Not meeting | 0.79 (0.62-1.02) | 0.88 (0.61-1.26) | 1.00 (0.78-1.29) | 0.99 (0.66-1.49) |
| Meeting | 0.57 (0.42-0.77) | 0.55 (0.35-0.88) | 0.79 (0.59-1.06) | 0.70 (0.42-1.16) |
|  | P-trend <0.001 | P-trend 0.016 | P-trend 0.120 | P-trend 0.180 |
| **eCRF**^†^ |  |  |  |  |
| Per MET | 0.89 (0.82-0.96) | 0.86 (0.77-0.97) | 0.90 (0.83-0.97) | 0.94 (0.83-1.06) |
| Quartile 1 | 1 (ref) | 1 (ref.) | 1 (ref.) | 1 (ref.) |
| Quartile 2 | 0.93 (0.70-1.22) | 0.91 (0.61-1.36) | 0.82 (0.62-1.09) | 1.22 (0.78-1.91) |
| Quartile 3 | 0.77 (0.57-1.03) | 0.64 (0.41-1.01) | 0.89 (0.67-1.19) | 0.88 (0.54-1.44) |
| Quartile 4 | 0.66 (0.48-0.91) | 0.63 (0.39-1.01) | 0.71 (0.52-0.95) | 0.66 (0.39-1.12) |
|  | P-trend 0.006 | P-trend 0.020 | P-trend 0.047 | P-trend 0.077 |
| Data are presented as hazard ratios (95% confidence intervals). CVD = cardiovascular disease; eCRF = estimated cardiorespiratory fitness; PA = physical activity ^*^PA adjusted for attained age, sex, BMI, CVD, smoking, alcohol, occupational status and CHA_2_DS_2_-VASc risk score ^†^eCRF adjusted for attained age, sex, CVD, smoking, alcohol, occupational status and CHA_2_DS_2_-VASc risk score. | | | | |

| **Supplementary table 3.** Baseline characteristics of HUNT3 participants without AF according to PA recommendations. | | | | |
| --- | --- | --- | --- | --- |
| Characteristic | | Inactive | Not meeting | Meeting |
| No. of participants | | 9 048 (21.4) | 15 264 (36.0) | 18 063 (42.6) |
| Sex | |  |  |  |
|  | Women | 3 810 (42.1) | 8 898 (58.3) | 10 397 (57.6) |
|  | Men | 5 238 (57.9) | 6 366 (41.7) | 7 666 (42.4) |
| Age (yrs) | | 50.4 ± 15.2 | 52.7 ± 15.2 | 49.7 ± 14.9 |
| Height (cm) | | 172.2 ± 9.3 | 170.1 ± 9.1 | 171.1 ± 8.9 |
| Weight (kg) | | 82.8 ± 16.3 | 79.2 ± 15.0 | 77.7 ± 14.2 |
| Waist (cm) | | 96.3 ± 12.9 | 93.9 ± 12.1 | 90.8 ± 11.4 |
| Body mass index (kg/m^2^) | | 27.9 ± 4.8 | 27.3 ± 4.4 | 26.4 ± 4.0 |
| Systolic BP (mmHg) | | 130.7 ± 17.7 | 130.5 ± 18.4 | 128.3 ± 17.6 |
| Diastolic BP (mmHg) | | 74.2 ± 11.3 | 73.6 ± 11.2 | 72.5 ± 10.9 |
| Resting heart rate (bpm) | | 70.0 ± 11.2 | 69.1 ± 11.1 | 66.1 ± 10.9 |
| eCRF (mL/kg/min) | | 34.8 ± 7.8 | 34.1 ± 7.4 | 38.9 ± 8.0 |
| eCRF (METs) | | 9.9 ± 2.2 | 9.7 ± 2.1 | 11.1 ± 2.3 |
| CHA_2_DS_2_VASc risk score | |  |  |  |
|  | Low-moderate | 7 484 (82.7) | 12 083 (79.2) | 15 368 (85.1) |
|  | High | 1 564 (17.9) | 3 181 (20.8) | 2 695 (14.9) |
| Smoking status | |  |  |  |
|  | Non-smoker | 5 771 (63.8) | 11 414 (74.8) | 14 633 (81.0) |
|  | Daily smoker | 2 567 (28.4) | 2 739 (17.9) | 2 052 (11.4) |
|  | Occasional | 710 (7.9) | 1 111 (7.3) | 1 378 (7.6) |
| Alcohol use^*^ | | 6 865 (75.9) | 11 800 (77.3) | 14 926 (79.3) |
| Hypertension† | | 3 463 (38.3) | 6 067 (39.8) | 5 812 (32.2) |
| Heart failure | | 64 (0.7) | 84 (0.6) | 78 (0.4) |
| Myocardial infarction | | 258 (2.6) | 428 (2.8) | 348 (1.9) |
| Stroke | | 233 (2.6) | 394 (2.6) | 305 (1.7) |
| Diabetes | | 380 (4.2) | 628 (4.1) | 568 (3.1) |
| Data are presented as means ± SD or No. (percentages).  BP = blood pressure; eCRF = estimated cardiorespiratory fitness; MET = metabolic equivalent; PA = physical activity, ^*^Alchohol use last 2 weeks, †Systolic BP ≥140 mmHg and/or diastolic BP ≥90 mmHg and/or use of antihypertensive medication. | | | | |

| **Supplementary table 4.** Hazard ratios with 95% CIs for all-cause mortality, CVD mortality, CVD morbidity and stroke according to AF status and PA levels. | | | | | | |
| --- | --- | --- | --- | --- | --- | --- |
|  |  | No AF | |  | AF | |
| Outcome | PA recommendations | HR* | 95% CI |  | HR* | 95% CI |
| All-cause mortality | Inactive | 1 (ref.) |  |  | 1.50 | 1.22-1.82 |
|  | Not meeting | 0.82 | 0.74-0.91 |  | 1.19 | 0.98-1.44 |
|  | Meeting | 0.67 | 0.59-0.75 |  | 0.90 | 0.70-1.15 |
|  |  |  |  |  |  |  |
| CVD mortality | Inactive | 1 (ref.) |  |  | 2.12 | 1.56-2.89 |
|  | Not meeting | 0.79 | 0.64-0.98 |  | 1.75 | 1.29-2.36 |
|  | Meeting | 0.65 | 0.51-0.83 |  | 1.14 | 0.76-1.71 |
|  |  |  |  |  |  |  |
| CVD morbidity | Inactive | 1 (ref.) |  |  | 1.67 | 1.35-2.06 |
|  | Not meeting | 0.90 | 0.81-1.00 |  | 1.67 | 1.38-2.01 |
|  | Meeting | 0.79 | 0.71-0.88 |  | 1.35 | 1.08-1.69 |
|  |  |  |  |  |  |  |
| Stroke | Inactive (ref.) | 1 (ref.) |  |  | 1.50 | 1.07-2.11 |
|  | Not meeting | 0.92 | 0.78-1.08 |  | 1.43 | 1.06-1.93 |
|  | Meeting | 0.77 | 0.65-0.92 |  | 0.99 | 0.67-1.47 |
| Data are presented as hazard ratios with 95% confidence intervals.  CVD = cardiovascular disease; eCRF = estimated cardiorespiratory fitness; PA = physical activity ^*^Adjusted for attained age, sex, body mass index, diabetes, hypertension, CVD, smoking, alcohol and occupational status. | | | | | | |

| **Supplemental table 5.** Hazard ratios with 95% CIs for all-cause mortality according to PA recommendations and eCRF in AF patients. | | | |
| --- | --- | --- | --- |
|  | All-cause mortality | | |
| PA | Model 2 + AF subtype |  | Model 2 + betablocker use |
| Inactive | 1 (ref.) |  | 1 (ref.) |
| Not meeting | 0.81 (0.52-1.27) |  | 0.88 (0.57-1.36) |
| Meeting | 0.66 (0.38-1.13) |  | 0.71 (0.41-1.22) |
| eCRF |  |  |  |
| Quartile 1 | 1.0 (ref.) |  | 1.0 (ref.) |
| Quartile 2 | 1.40 (0.87-2.24) |  | 1.33 (0.82-2.13) |
| Quartile 3 | 0.96 (0.55-1.69) |  | 0.96 (0.55-1.67) |
| Quartile 4 | 0.78 (0.44-1.39) |  | 0.77 (0.43-1.37) |
| Data are presented as hazard ratios (95% confidence intervals).  CVD = cardiovascular disease; eCRF = estimated cardiorespiratory fitness; PA = physical activity ^*^Model 2 adjusted for sex, age by including attained age as the time scale, body mass index, CVD, smoking, alcohol and occupational status. AF subtype (paroxysmal, persistent, permanent), betablocker use at baseline. | | | |


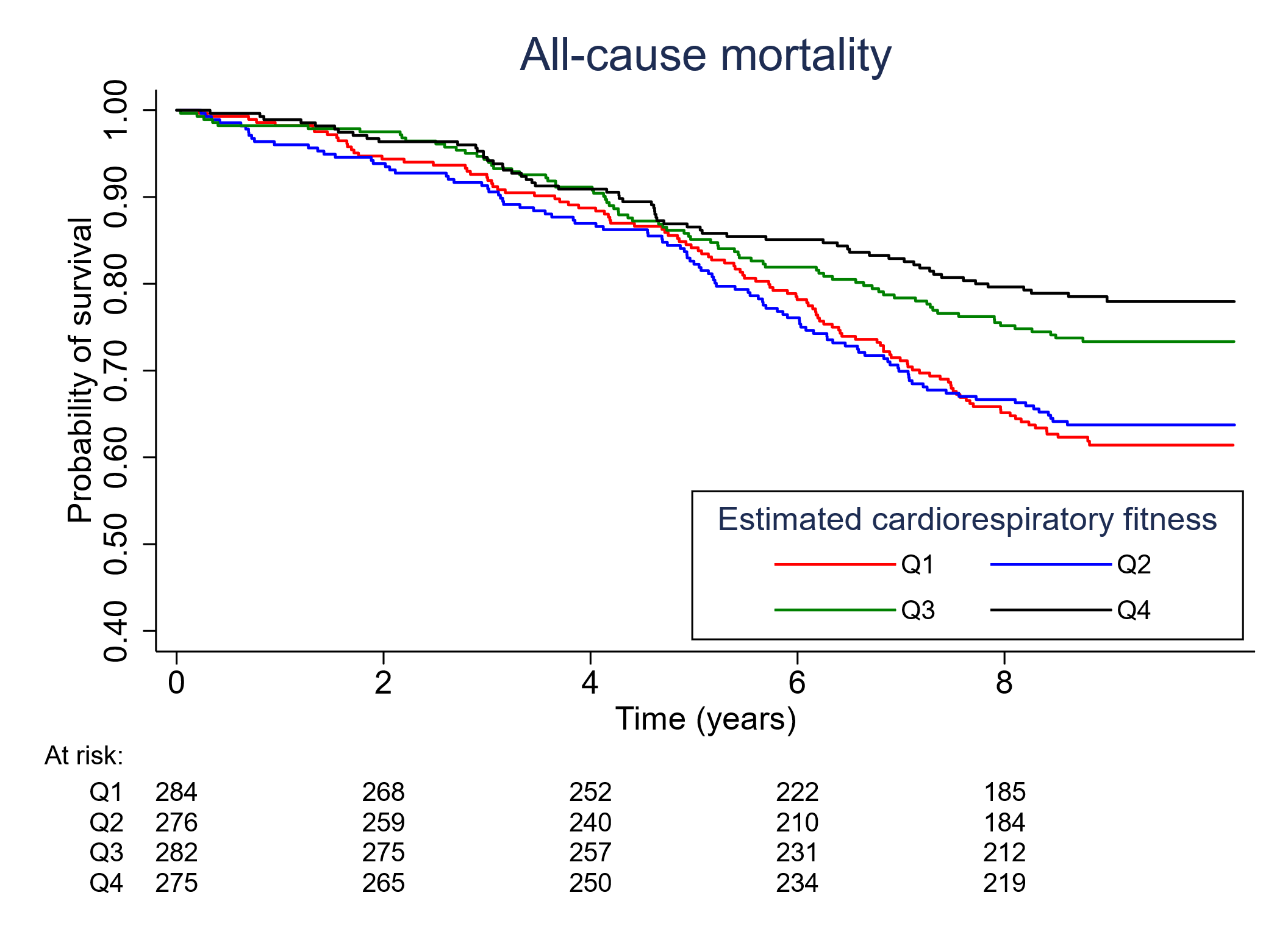

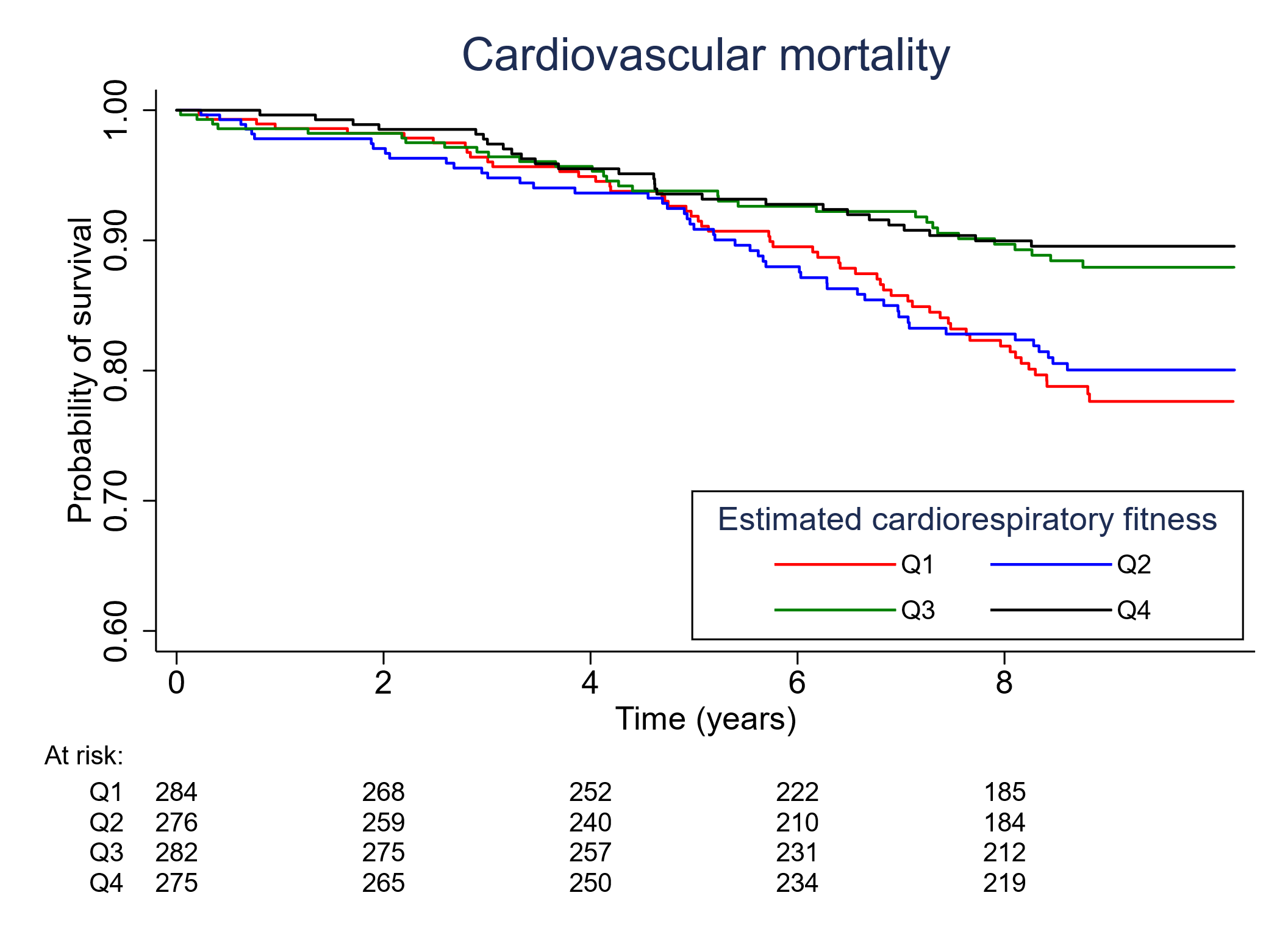


**Supplemental figure 1.** The Kaplan-Meier curves illustrates the probability of survival for all-cause mortality and CVD mortality within age- and sex-specific quartiles of eCRF. The at-risk table displays the numbers at risk for each time point.


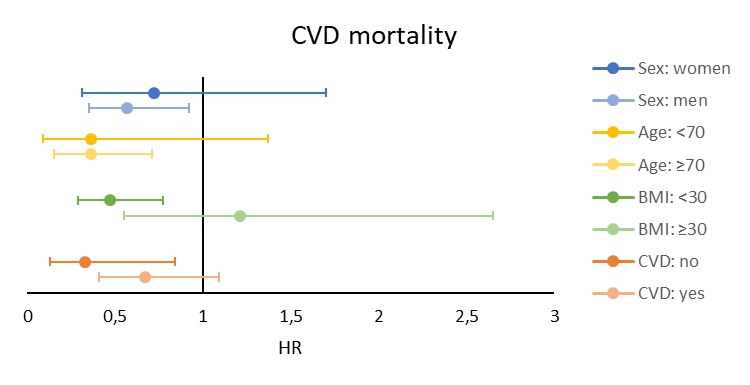


B

C


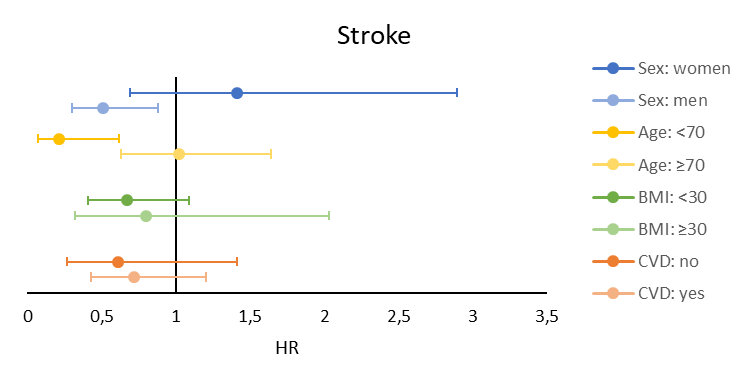


D


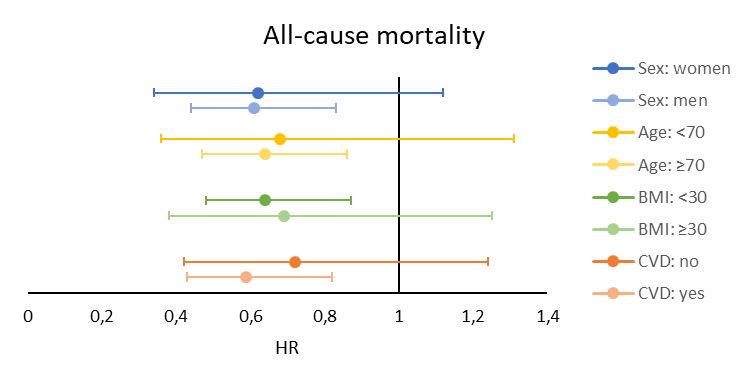


A

**Supplemental figure 2.** Association between PA and all-cause mortality, CVD mortality, CVD morbidity and stroke within subgroups. Hazard ratios with 95% CI`s represents participants meeting PA recommendations compared to not meeting (reference). Adjusted for age, sex, BMI, CVD, smoking, alcohol and occupational status, except from the variable stratified on.


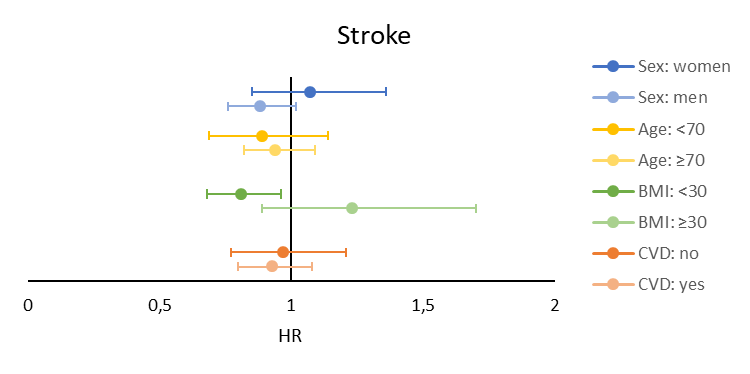

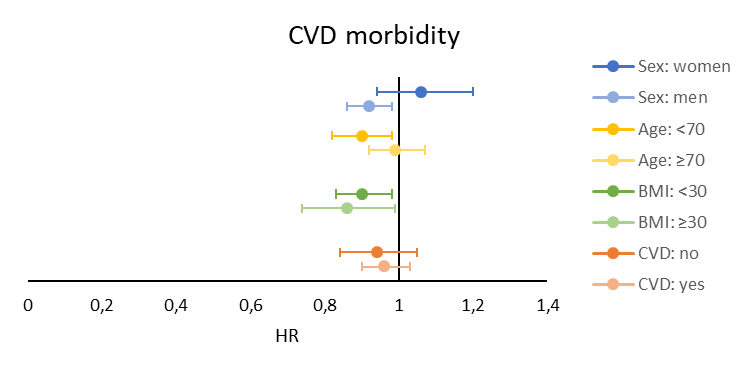

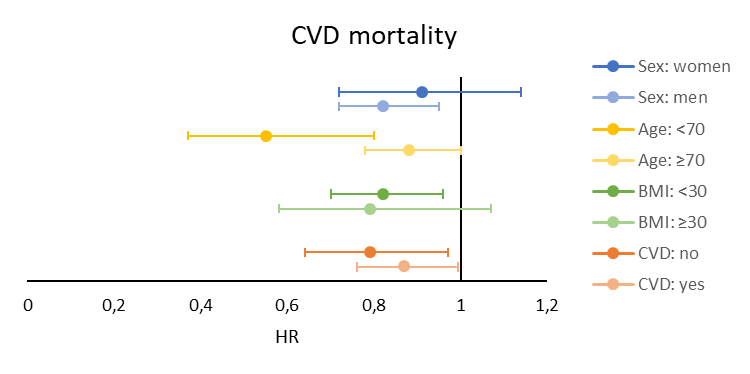

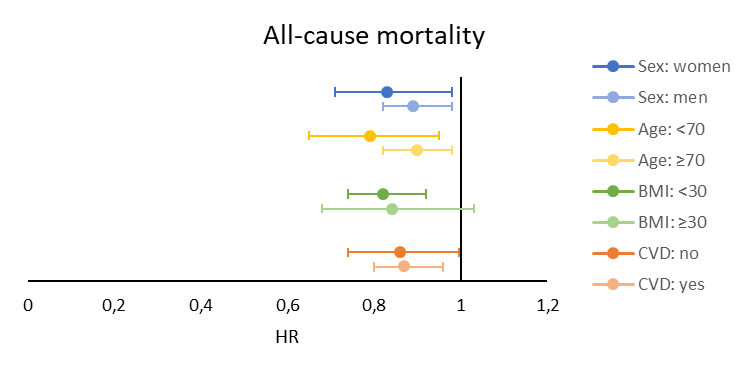


B

C

D

A

**Supplemental figure 3.** Association between eCRF and all-cause mortality, CVD mortality, CVD morbidity and stroke within subgroups. Hazard ratios with 95% CI`s presented is per 1-MET higher eCRF. Adjusted for age, sex, CVD, smoking, alcohol and occupational status, except from the variable stratified on.


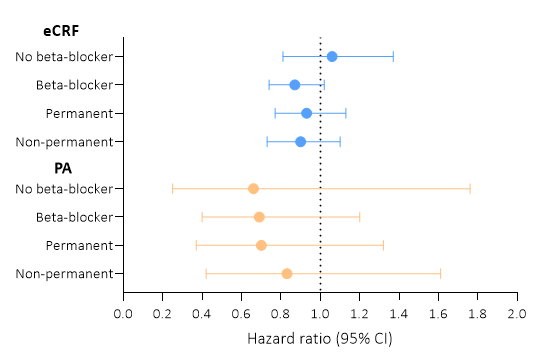


**Supplemental figure 4.** Association between PA and eCRF, respectively, and all-cause mortality stratified by beta-blocker use and permanent vs. non-permanent AF at baseline. Hazard ratios with 95% CI`s represents meeting vs. not meeting general PA recommendations (reference) and per 1-MET higher eCRF. Adjusted for age, sex, CVD, BMI, smoking, alcohol and occupational status. Data from a subgroup of 477 with available information on stratification variables.
